# Mechanical tensile properties of the anterolateral ligament

**DOI:** 10.1186/s40634-015-0023-3

**Published:** 2015-04-02

**Authors:** Martin Zens, Matthias J Feucht, Johannes Ruhhammer, Anke Bernstein, Hermann O Mayr, Norbert P Südkamp, Peter Woias, Philipp Niemeyer

**Affiliations:** Albert-Ludwigs-University Freiburg, Department of Microsystems Engineering (IMTEK), Design of Microsystems, Georges-Koehler-Allee 102, Freiburg i. Brsg., 79110 Germany; University Medical Center Freiburg, Department of Orthopaedics and Trauma Surgery, Hugstetter Str. 55, Freiburg i. Brsg., 79106 Germany

**Keywords:** Anterolateral ligament, ALL, Biomechanics, Load to failure, Tensile testing, Mechanical properties, Histology

## Abstract

**Background:**

In a noticeable percentage of patients anterolateral rotational instabilities (ALRI) remain after an isolated ACL reconstruction. Those instabilities may occur due to an insufficiently directed damage of anterolateral structures that is often associated with ACL ruptures. Recent publications describe an anatomical structure, termed the anterolateral ligament (ALL), and suggest that this ligament plays a significant role in the pathogenesis of ALRI of the knee joint. However, only limited knowledge about the biomechanical characteristics and tensile properties of the anterolateral ligament exists.

**Methods:**

The anterolateral ligament was dissected in four fresh-frozen human cadaveric specimens and all surrounding tissue removed. The initial length of the anterolateral ligament was measured using a digital caliper. Tensile tests with load to failure were performed using a materials testing machine. The explanted anterolateral ligaments were histologically examined to measure the cross-sectional area.

**Results:**

The mean ultimate load to failure of the anterolateral ligament was 49.90 N (± 14.62 N) and the mean ultimate strain was 35.96% (± 4.47%). The mean length of the ligament was 33.08 mm (± 2.24) and the mean cross-sectional area was 1.54 *mm*^2^ (± 0.48 *mm*^2^). Including the areal measurements the maximum tension was calculated to be 32.78 $\frac {N}{{mm}^{2}}$ (± 4.04 $\frac {N}{{mm}^{2}}$).

**Conclusions:**

The anterolateral ligament is an anatomical structure with tensile properties that are considerably weaker compared to other peripheral structures of the knee. Knowledge of the anterolateral ligament’s tensile strengths may help to better understand its function and with graft choices for reconstruction procedures.

## Background

Traumatic injuries to the anterior cruciate ligament (ACL) are one of the most common reasons for clinical presentation in sports medicine. Its surgical repair follows standard procedures which evolved over several decades. However, in a noteworthy percentage of patients anterolateral rotational instabilities (ALRI) remain following state-of-the-art surgery. Reasons for this observation are discussed in current literature and several research groups suggest that injury to anterolateral structures of the knee joint may result in ALRI. Baker et al. (1983); Hughston et al. (1976); Tanaka et al. (2012); Terry et al. (1993), Wroble et al. (1993) in an anatomical study, Claes et al. (2013) recently investigated a ligamentous structure that had primarily been described by Segond (1879) in 1879. This structure, termed anterolateral ligament (ALL), connects the lateral epicondylus of the femur with the anterolateral proximal tibia and was found in 40 of 41 examined specimen. Those findings were confirmed by other research groups. Caterine et al. (2014); Dodds et al. (2014); Helito et al. (2013); Parsons et al. (2015); Zens et al. (2014) independently, various authors suggested that the ALL may be a significant stabilizer in the knee. Reconstruction was proposed to be necessary to avoid or diminish ALRI, especially for revision cases and primary cases with gross positive pivot-shifts (Claes et al. 2013; Helito et al. 2014, 2013; Rezansoff et al. 2014, Sonnery-Cottet et al. 2014). However, before implying such a necessity a thorough biomechanical analysis ought to be conducted. Knowledge about the ligaments tensile properties may serve a better understanding of the ligaments behavior, allows a fact-based assessment of its possible contribution to ALRI and is essential for the selection of a suitable transplant and reconstruction technique. Up until now only very few studies have been published regarding the mechanical properties of the ALL. All of those studies have investigated the length changes of the ALL during passive knee motion. The purpose of this study was to determine typical mechanical properties of the ALL through tension testing. No previous study has measured the maximum load to failure of the ALL. By comparing this data with values previously determined for other ligaments and considering the absolute values, such as ultimate tensile stress, ultimate strain and Young’s modulus, a better evaluation of the ligaments significance can be given. Furthermore, possible reconstruction grafts with similar mechanical features can be identified.

## Methods

### Dissection technique

**Figure 1:**
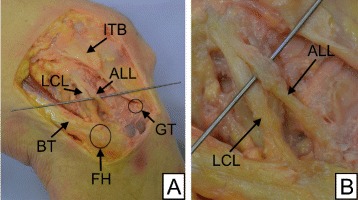
**Dissected ALL.** **A**: Dissected anterolateral ligament (ALL) with surrounding structures in a right knee; ITB: iliotibial band, LCL: lateral collateral ligament, GT: Gerdy’s tubercle, BT: biceps tendon, FH: fibular head. **B**: Detailed view of the dissected ALL.

Four fresh-frozen human cadaveric knees were included in this study (3 male, 1 female). The cadaveric knees were obtained from the Institut of Anatomy, Friedrich-Alexander-University of Erlangen-Nuremberg, with written consent from all donors and in accordance with ethical approval of the University of Freiburg (Ref. 45/15). The mean age of the specimen was 86.5 ± 1.7. None of the studied knees had undergone significant surgery or showed signs of bone deformity. Before testing, the specimens were thawed for 48 hours. All tests were performed at room temperature and the specimens were constantly kept moist with saline solution. Initially the ALL was carefully dissected using a standardized procedure described by Claes et al. (2013). This was done by a single investigator with the knee in 90° of flexion. Bony landmarks of the ALL, being the lateral epicondyle, the fibular head and Gerdy’s tubercle, were identified through palpation. A hand sized, rectangular cuteness flap was removed above this area. After removal of subcutaneous fat the iliotibial tract, extensor apparatus and the short head of the biceps femoris were displayed. Subsequently, the iliotibial tract was cut through approximately 60 mm proximal of the lateral epicondyle and delicately released to its tibial attachment by cutting the Kaplan fibers attached to the lateral intermusculare septum and the lateral retinaculum. Following this step the lateral collateral ligament (LCL) was identified and the distal tibia rotated in interior direction, thus allowing an identification of the ALL. In all four specimen a ligamentous structure, as described in previous publications, was identified connecting the lateral femoral epicondyle and the anterolateral proximal tibia (Figure 1). The initial length *L*_0_ of the ALL was measured in 0° flexion using a digital caliper and documented. Afterwards the ALL was carefully isolated and undermined with a surgical vessel loop. Tibia and femur were thoroughly fixated in two hollow aluminum cylinders that were used to mount the knee joints in the materials testing machine. This step was succeeded by a radical removal of all connecting structures between thigh and lower leg, except the ALL, leaving the anterolateral ligament as the only connecting tissue bridge.

### Testing procedure

Uniaxial tensile failure tests were conducted on a servohydraulic materials testing machine (Zwick/Roell Amsler HC 10, Zwick/Roell AG, Ulm, Germany) at an extension rate of 0.5 *mm*/*s*. The fiber course of the ALL was in line with the direction of force thereby simulating a worst-case scenario. All four specimen were tested without preconditioning cycles. The stop criterion was a relative drop in force of 90%. A force-distance curve, ultimate load to failure (in *N*) and ultimate extension distance (in *mm*) were recorded for each specimen. The sampling rate was set to 100 Hz. Stress-strain curves, ultimate tensile stress (in *N*/*mm*^2^), ultimate strain (in %) and Young’s modulus at 20% strain were calculated by taking the initial length *L*_0_ and the cross-sectional area *A*_0_ into account.

### Cross-sectional area measurement

Accurate cross-sectional measurements of the ALL were performed by obtaining and dimensioning histological cross-sectional cuts. For this purpose the torn ALL of each cadaveric knee was explanted after tensile testing and quick-frozen in liquid isopentane (*T*<−80°C). Tissue Freezing Medium®; (Leica Biosystems Nussloch GmbH, Nussloch, Germany) was used to embed the quick-frozen ALL. Cuts along approximately five equidistant sections of each ALL were fixated on a specimen holder. A Leica CM3050 S cryostat (Leica Biosystems Nussloch GmbH, Nussloch, Germany) was utilized for this task. Following fixation, representative cuts of each section were stained with HE and Giemsa. AxioVision (Carl Zeiss AG, Oberkochen, Germany) software was used to acquire images and measure the cross-sectional area. The mean was determined from cuts of the five equidistant sections.

### Statistical analysis

Measurements were plotted and analyzed using OriginPro®; 9.0 (OriginLab Corp., Northampton, MA, USA). Correlations between initial length, cross-sectional area, ultimate load to failure, ultimate stress and ultimate distance were revealed by calculating correlation coefficients. Subsequently, Student’s t-tests were performed to test significance. Significance was set at P < 0.05.

## Results

**Figure 2:**
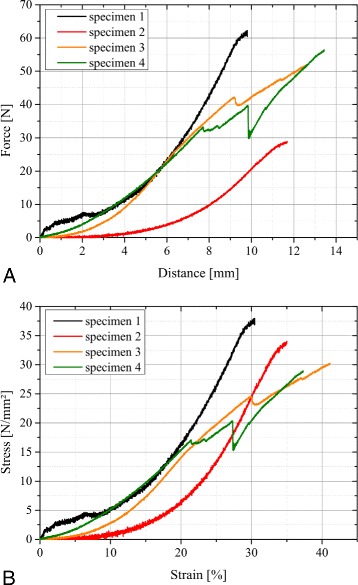
**Measurement results.** **A**: Force-distance-curve directly recorded using the materials testing machine; **B**: Stress-strain-curve calculated by taking relative length changes and cross-sectional areas into account.

All four specimen showed an interligamentous failure at approximately one third of the ALL’s length distal from the femorial insertion site. A force-distance curve with an average of 2,399 (± 318) data points was recorded for each specimen. The resulting curves are shown in Figure 2A. The mean ultimate load to failure was 49.90 N ± 14.62 N and the mean ultimate extension distance was 11.89 mm ± 1.56 mm. Hence, resulting in a mean extensional stiffness of 2.60 N ± 0.93 N.

In order to calculate stress-strain curves the initial length *L*_0_ and the average cross-sectional area *A*_0_ per ligament were included. Whereas the initial length was measured prior to the tensile tests, the cross-sectional area was determined by measuring five cuts of each ALL and calculating their average. The mean initial length was 33.1 mm ± 2.2 mm and the mean cross-sectional area 1.54 *mm*^2^± 0.48 *mm*^2^. Dividing force by the cross-sectional area and distance by the initial length results in the stress-strain curves displayed in Figure 2B. The mean ultimate stress was 32.78 $\frac {N}{{mm}^{2}} \pm $ 4.04 $\frac {N}{{mm}^{2}}$ and the mean ultimate strain 35.96% ± 4.47%. A Young’s modulus was calculated at 20% strain with a mean value of 1.20 $\frac {N}{{mm}^{2}} \pm $ 0.44 $\frac {N}{{mm}^{2}}$. Specimen 3 and 4 show a partial failure between 25 and 30% strain prior to ultimate failure of the ligament. Statistical analysis revealed a significant correlation between ultimate load to failure and cross-sectional area (r = 0.897; P < 0.05) as well as ultimate stress and ultimate distance (r = -0.990; P < 0.01). The initial length of the ALL has no significant effect on the ultimate strain (r = -0.126; P > 0.1). All measurement results are summarized in Table 1.

**Table 1:** **Measurement results of tensile tests of the anterolateral ligament**

**Parameter**	**Specimen 1**	**Specimen 2**	**Specimen 3**	**Specimen 4**	**Mean**	**SD**
Data points [*n*]	1,961	2,379	2,569	2,686	2,399	± 318
Length *L*_0_ [*mm*]	32.2	33.4	30.7	36.0	33.1	± 2.2
Cross-sectional area *A*_0_ [ *m* *m* ^2^]	1.64	0.85	1.72	1.95	1.54	± 0.48
Max. force [*N*]	62.21	28.88	52.01	56.48	49.90	± 14.62
Max. distance [*mm*]	9.80	11.69	12.62	13.43	11.89	± 1.56
Max. stress [$\frac {N}{{mm}^{2}}$]	37.93	33.98	30.24	28.96	32.78	± 4.04
Max. strain [ *%*]	30.43	34.99	41.12	37.31	35.96	± 4.47
Extensional stiffness [*N*]	3.99	2.04	2.24	2.15	2.60	± 0.93
Young’s modulus (20%) [$\frac {N}{{mm}^{2}}$]	1.80	1.14	1.13	0.74	1.20	± 0.44

**Figure 3:**
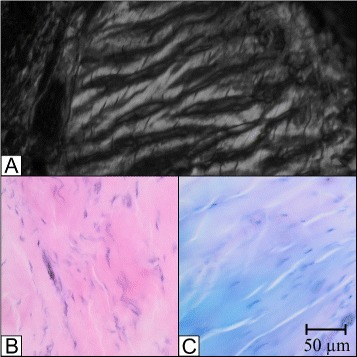
**Histology of the ALL.** Different histological illustrations of the ALL; **A**: Polarization with crimping; **B**: HE stain; **C**: Giemsa stain.

Besides cross-sectional area measurements the histological cuts were further investigated regarding the composition of the anterolateral ligament. Using polarization microscopy a unique crimping pattern was found for the ALL. This proves the existence of a ligamentous structure. Figure 3A shows the crimping of the ALL along with detailed images of HE stained (Figure 3B) and Giemsa stained (Figure 3C) histological samples.

## Discussion

The purpose of this study was to determine the tensile mechanical properties of the anterolateral ligament. Terry et al. (1993) do not consider the ALL to be a distinct ligamentous structure and use this term for a synergistically acting combination of superficial, deep and capsulo-osseous layers of the ilitibial tract. The results of this study are consistent with previous studies (Claes et al. 2013; Helito et al. 2013) and show a clearly distinguishable fibre course with evidently defined insertion sites for the four knees investigated. Hence, the ALL can be considered as a self-contained anatomical structure. However, findings and supposed interaction of iliotibial band layers suggest that not a single stabilizer, but rather a complex system of anterolateral structures is responsible for rotational stability with the ALL being one element of this system. Further actors of this system are the iliotibial band and the anterolateral capsule. Kittl et al. (2014) in the present study the ALL is investigated as an isolated structure. Clinically however, the ALL and its tensile properties have to be viewed in context with synergistically acting anatomical elements. Typically severe injuries will result in a traumatic destruction of the anterolateral capsule and the ALL. Isolated ALL ruptures are very unlikely, which lessens the clinical relevance of the low absolute values for load of failure (De Beule et al. 2014; Sonnery-Cottet et al. 2015).

Current publications already describe and discuss ALL reconstruction techniques without sufficient knowledge of the fundamental properties. Previous studies addressing the biomechanical properties of the anterolateral ligament have their focus on length changes during physiological motion of the knee. Claes et al. (2013); Dodds et al. (2014); Helito et al. (2013, 2014); Kittl et al. (2014); Parsons et al. (2015) whereas the information about length change is important for reconstruction techniques, tensile properties allow a better assessment of a ligament’s significance. Dodds et al. (2011) this is the first study investigating the tensile properties of the ALL. The mean ultimate tension of 32.78 $\frac {N}{mm^{2}}$ (± 4.04 $\frac {N}{mm^{2}}$) shows a value that is comparable to other ligaments, e.g. ACL 37.80 $\frac {N}{mm^{2}}$ (± 3.80 $\frac {N}{mm^{2}}$) and MCL 38.60 $\frac {N}{mm^{2}}$ (± 4.80 $\frac {N}{mm^{2}}$). (Noyes et al. 1984; Moon et al. 2006; Quapp and Weiss 1998) however, because of the small cross-sectional area of the anteroateral ligament the ultimate load to failure of 49.90 N is only a fraction of values measured for ACL (1,725 N ± 269 N), (MCL 1,107 N ± 126 N) and PCL (1,051 N ± 237 N). The iliotibial tract displays a considerably lower ultimate tension of 19.1 N (± 2.9 N), but because of its dimensions a 15 times higher ultimate load to failure (769 N ± 99 N). All values reported above are based on findings described for a group of young specimen (ACL, IT: mean age 27 years; MCL, PCL: mean age unknown). Ligaments with a similar order of magnitude are the LCL (309 N ± 91 N) and the popliteofibular ligament complex (PFLC) (186 N ± 65 N)). The study for LCL and PFLC was performed on older specimen with a mean age of approximately 70 years. Table 2 gives an overview about tensile properties of various anatomic structures previously described. Table 2 gives an overview about tensile properties of various anatomic structures previously described.

**Table 2:** **Ultimate tension and ultimate load to failure of the ALL in comparison to other ligaments and possible grafts**

**Structure**	**Ultimate tension [MPa]**	**Ultimate load to**	**Relation to ALL**
		**failure [N]**	**(load to failure) [%]**
**ALL**	**32.78 ± 4.04**	**49.90 ± 14.62**	**100**
ACL^1^ (Noyes et al. 1984)	37.80 ± 3.80	1,725 ± 269	3,457
PCL^2^ (Noyes et al. 1984; Race and Amis 1994)	35.90 ± 15.20	739 - 1,627	1,481
MCL^3^ (Moon et al. 2006; Quapp and Weiss 1998)	38.60 ± 4.80	1,107 ± 126	2,219
Distal sMCL^3^ (Wijdicks et al. 2010)	-	557 ± 55	1,116
Proximal sMCL^3^ (Wijdicks et al. 2010)	-	88 ± 36	176
POL^4^ (Wijdicks et al. 2010)	-	256 ± 30	513
Deep MCL^3^ (Wijdicks et al. 2010)	-	101 ± 10	202
LCL^5^ (Sugita and Amis 2001)	-	309 ± 91	619
PFLC^6^ (Sugita and Amis 2001)	-	186 N ± 65	373
ITB^7^ (Noyes et al. 1984)	19.1 ± 2.9	769 ± 99	1,541
Fascia lata (Noyes et al. 1984)	78.7 ± 4.6	628 ± 35	1,259
Semitendinosus (Noyes et al. 1984)	88.5 ± 5.0	1,216 ± 50	2,437
Gracilis (Noyes et al. 1984)	111.5 ± 4.0	838 ± 30	1,679

^1^Anterior cruciate ligament, ^2^posterior cruciate ligament, ^3^(superficial) medial collateral ligament, ^4^posterior oblique ligament, ^5^lateral collateral ligament, ^6^popliteofibular ligament complex, ^7^iliotibial band.

Noyes and Grood (1976), as well as Woo et al. (1991) have shown in separate studies, that the ultimate load to failure of the ACL in younger patients is 2.5 times higher than in older patients. In case a similar trend applies to the anterolateral ligament a value around 125 N may be possible. Wang et al. (2006) report an age-dependent modification of insertion sites leading to a decreasing load to failure. Furthermore this study has shown a strong correlation between the cross-sectional area, thus representing the dimensions of the ALL, and the ultimate load to failure. The size of the ALL is expected to reduce with age, hence resulting in a decreasing maximum load. Other studies (Matthews et al. 1968; Moon et al. 2006; Viidik and Lewin 1966; Woo et al. 1986) have investigated how freezing of cadavers effects the tensile properties of ligaments. The results showed a concurrent worsening of the measured properties, thus implying that the *in vivo* features of the anterolateral ligament are rather underestimated in this study. Even if all possible limitations are taken into account the tensile properties the ALL are significantly lower than those of primary stabilizers of the knee, such as ACL, LCL and MCL. Further investigation is necessary to definitely conclude how the ALL contributes to ALRI. Based on the failure properties discovered in this study and recognizing the limitations of testing cadaveric tissues the ALL is to be considered a structure that acts synergistically with primary stabilizers, such as LCL, ACL and iliotibial band to support the knee. Furthermore, recommendations for reconstruction grafts can be proposed based on those findings. Claes at al. (2013) suggest an autologous graft of the gracilis tendon for an anatomical repair of the ALL. Notwithstanding, all autologous and artificial grafts choices displayed in Table 2 provide a sufficient load to failure to replace the ALL, but the ultimate tension of the gracilis tendon matches the ALL the least. Based on the preliminary data of this study other possible graft options, such as iliotibial band (ITB) or semitendinosus tendon split-offs appear more suitable. Besides choosing an appropriate graft for reconstruction of the ALL the development of a suitable technique is equally important. Lemaire et al. (1980) presented a surgical method coping with anterolateral instabilities using an ITB split-off. This approach has been adapted recently by Wagner et al. (2014). Essentially, these techniques use a ITB split-off. This split-off is shuttled underneath the LCL to create a dynamic stabilization of the joint in an anatomic course that is consistent with the axis of the ALL. This ITB-based approach is found in a similar manner in other techniques. Carson (1985), Österman et al. 1993, Wolff et al. (2005) approaches based on the semitendinosus tendon to address ALRI have also been discussed in literature (Ulmer et al. 2006; Zantop and Petersen 2010).

This study has several limitations. Apart from limitations that are generally associated with cadaveric measurements, such as post-mortem degeneration of tissue, age-dependency and effects of cadaver freezing, this study is based on measurements of only four cadaveric knee joints. However, the data of those four measurements shows little variances and is very consistent. Considering the fact that absolutely no data regarding the tensile properties of the ALL exists, but reconstruction methods are already being developed, this preliminary data suggests that further investigation of the biomechanical and especially tensile properties of the ALL is necessary, before continuing with the development of surgical techniques. Another limitation of this study is the determination of the cross-sectional area of explanted ligaments after load to failure testing. Tearing causes a constriction of the ligaments, which underestimates the cross-sectional area prior to testing. Furthermore the uniaxial direction of force tested in the measurement setup does not coincide with the physiological axes of the anterolateral ligament as a rotational stabilizer.

## Conclusion

The anterolateral ligament is an anatomical structure with an ultimate load to failure of 49.90 N and an ultimate tension of 32.78 $\frac {N}{mm^{2}}$. Based on the findings of this study a definite conclusion regarding the significance of the ALL cannot be given, but our data strongly suggests that the ALL is not a primary stabilizer of the knee joint as the tensile strengths are significantly lower than those of primary stabilizers. Further fundamental biomechanical research is necessary before starting to clinically reconstruct the ALL.

## References

[CR1] Arthrex, I (2013) Anterolateral Ligament Reconstruction using SwiveLock. Video. [last downloaded 08/02/2014]. https://www.arthrex.com/resources/video/JOVv2r2KoE-v7gFCU0pqVw/anterolateral-ligament-reconstruction-using-swivelock.

[CR2] Baker CL, Norwood LA, Hughston JC (1983). Acute posterolateral rotatory instability of the knee. J Bone Joint Surgery Am Volume.

[CR3] Carson WG (1985). Extra-articular reconstruction of the anterior cruciate ligament: lateral procedures. Orthopedic Clinics North Am.

[CR4] Caterine, S, Litchfield R, Johnson M, Chronik B, Getgood A (2014) A cadaveric study of the anterolateral ligament: re-introducing the lateral capsular ligament. Knee Surgery, Sports Traumatology, Arthroscopy: Official J ESSKA. doi:10.1007/s00167-014-3117-z. 10.1007/s00167-014-3117-z 24929656

[CR5] Claes S, Vereecke E, Maes M, Victor J, Verdonk P, Bellemans J (2013). Anatomy of the anterolateral ligament of the knee. J Anatomy.

[CR6] De Beule J, Vandenneucker H, Claes S, Bellemans J (2014). Can anterior cruciate ligament reconstruction be performed routinely in day clinic?. Acta Orthopædica Belgica.

[CR7] Dodds AL, Gupte CM, Neyret P, Williams AM, Amis AA (2011). Extra-articular techniques in anterior cruciate ligament reconstruction: a literature review. J Bone Joint Surgery. Br Volume.

[CR8] Dodds AL, Halewood C, Gupte CM, Williams A, Amis AA (2014). The anterolateral ligament: Anatomy, length changes and association with the Segond fracture. Bone Joint J.

[CR9] Helito CP, Demange MK, Bonadio MB, Tirico LEP, Gobbi RG, Pecora JR, Camanho GL (2013). Anatomy and Histology of the Knee Anterolateral Ligament. Orthopaedic J Sports Med.

[CR10] Demange MK, Bonadio MB, Tirico LEP, Gobbi RG, Pecora JR, Camanho GL, Helito, C P (2014). Radiographic landmarks for locating the femoral origin and tibial insertion of the knee anterolateral ligament. Am J Sports Med.

[CR11] Hughston JC, Andrews JR, Cross MJ, Moschi A (1976). Classification of knee ligament instabilities. Part II. The lateral. J Bone Joint Surg Am.

[CR12] Kittl, C, Halewood C, Stephen JM, Gupte CM, Weiler A, Williams A, Amis AA (2014) Length change patterns in the lateral extra-articular structures of the knee and related reconstructions. Am J Sports Med 0363546514560993. doi:10.1177/0363546514560993. 10.1177/0363546514560993 25540293

[CR13] Lemaire M, Combelles F (1980). Technique actuelle de plastie ligamentaire pour rupture ancienne du ligament croise anterieur. Rev Chir Orthop.

[CR14] Matthews LS, Ellis D (1968). Viscoelastic properties of cat tendon: Effects of time after death and preservation by freezing. J Biomechanics.

[CR15] Moon DK, Woo SL-Y, Takakura Y, Gabriel MT, Abramowitch SD (2006). The effects of refreezing on the viscoelastic and tensile properties of ligaments. J Biomechanics.

[CR16] Noyes FR, Grood ES (1976). The strength of the anterior cruciate ligament in humans and Rhesus monkeys. J Bone Joint Surgery. Am Volume.

[CR17] Noyes FR, Butler DL, Grood ES, Zernicke RF, Hefzy MS (1984). Biomechanical analysis of human ligament grafts used in knee-ligament repairs and reconstructions. J Bone Joint Surgery. Am Volume.

[CR18] Österman, K, Kujala UM, Kivimäki J, Österman H (1993) The MacIntosh lateral substitution reconstruction for anterior cruciate deficiency. Int Orthopaedics 17(4). doi:10.1007/BF00194183. 10.1007/BF00194183 8407037

[CR19] Parsons, EM, Gee AO, Spiekerman C, Cavanagh PR (2015) The biomechanical function of the anterolateral ligament of the knee. Am J Sports Med. doi:10.1177/0363546514562751. 10.1177/0363546514562751 PMC4708263 25556221

[CR20] Quapp KM, Weiss JA (1998). Material Characterization of Human Medial Collateral Ligament. J Biomech Eng.

[CR21] Race A, Amis AA (1994). The mechanical properties of the two bundles of the human posterior cruciate ligament. J Biomechanics.

[CR22] Rezansoff, AJ, Caterine S, Spencer L, Tran MN, Litchfield RB, Getgood AM (2014) Radiographic landmarks for surgical reconstruction of the anterolateral ligament of the knee. Knee Surgery, Sports Traumatology, Arthroscopy: Official J ESSKA. doi:10.1007/s00167-014-3126-y. 10.1007/s00167-014-3126-y 24934928

[CR23] Segond P (1879). Recherches Cliniques et Expérimentales sur les épanchements Sanguins du Genou Par Entorse. Publications du Progrès médical.

[CR24] Sonnery-Cottet B, Archbold P, Rezende FC, Neto AM, Fayard J-M, Thaunat M (2014). Arthroscopic Identification of the Anterolateral Ligament of the Knee. Arthroscopy Tech.

[CR25] Sonnery-Cottet, B, Thaunat M, Freychet B, Pupim BHB, Murphy CG, Claes S (2015) Outcome of a Combined Anterior Cruciate Ligament and Anterolateral Ligament Reconstruction Technique With a Minimum 2-Year Follow-up. Am J Sports Med 0363546515571571. doi:10.1177/0363546515 571571. 10.1177/0363546515571571 25740835

[CR26] Sugita T, Amis AA (2001). Anatomic and Biomechanical Study of the Lateral Collateral and Popliteofibular Ligaments. Am J Sports Med.

[CR27] Tanaka M, Vyas D, Moloney G, Bedi A, Pearle AD, Musahl V (2012). What does it take to have a high-grade pivot shift?. Knee Surgery, Sports Traumatology, Arthroscopy: Official J ESSKA.

[CR28] Terry GC, Norwood LA, Hughston JC, Caldwell KM (1993). How iliotibial tract injuries of the knee combine with acute anterior cruciate ligament tears to influence abnormal anterior tibial displacement. Am J Sports Med.

[CR29] Ulmer MG, Rose T, Imhoff AB (2006). Bandverletzungen Am Kniegelenk - Teil II. Orthopädie und Unfallchirurgie up2date - 1, vol. 1.

[CR30] Viidik A, Lewin T (1966). Changes in Tensile Strength Characteristics and Histology of Rabbit Ligaments Induced by Different Modes of Postmortal Storage. Acta Orthopaedica.

[CR31] Wagner M, Weiler A (2014). Anterolaterale Stabilisierung. Arthroskopie.

[CR32] Wang I-NE, Mitroo S, Chen FH, Lu HH, Doty SB (2006). Age-dependent changes in matrix composition and organization at the ligament-to-bone insertion. J Orthopaedic Research: Official Publ Orthopaedic Res Soc.

[CR33] Wijdicks CA, Ewart DT, Nuckley DJ, Johansen S, Engebretsen L, Laprade RF (2010). Structural properties of the primary medial knee ligaments. Am J Sports Med.

[CR34] Wolff D, Bidermann T, Hempfling H, Bühren V (2005). Die extraartikuläre kniestabilisierung nach macintosh-eine alternative bei fehlgeschlagenem vorderen kreuzbandersatz?. Zentralblatt für Chirurgie.

[CR35] Woo SL-Y, Hollis JM, Adams DJ, Lyon RM, Takai S (1991). Tensile properties of the human femur-anterior cruciate ligament-tibia complex: The effects of specimen age and orientation. Am J Sports Med.

[CR36] Woo SL-Y, Orlando CA, Camp JF, Akeson WH (1986). Effects of postmortem storage by freezing on ligament tensile behavior. J Biomechanics.

[CR37] Wroble RR, Grood ES, Cummings JS, Henderson JM, Noyes FR (1993). The role of the lateral extraarticular restraints in the anterior cruciate ligament-deficient knee. Am J Sports Med.

[CR38] Zantop T, Petersen W (2010). [Modified Larson technique for posterolateral corner reconstruction of the knee]. Operative Orthopädie und Traumatologie.

[CR39] Zens M, Ruhhammer J, Goldschmidtboeing F, Woias P, Feucht MJ, Mayr HO, Niemeyer P (2014). A New Approach to Determine Ligament Strain Using Polydimethylsiloxane Strain Gauges: Exemplary Measurements of the Anterolateral Ligament. J Biomech Eng.

